# Psychosocial Support for Healthcare Workers During the COVID-19 Pandemic

**DOI:** 10.3389/fpsyg.2020.01960

**Published:** 2020-08-11

**Authors:** Jack Tomlin, Bryan Dalgleish-Warburton, Gary Lamph

**Affiliations:** ^1^Department of Forensic Psychiatry, University of Rostock, Rostock, Germany; ^2^School of Community Health & Midwifery, University of Central Lancashire, Preston, United Kingdom

**Keywords:** COVID-19, Corona virus, psychosocial support, interventions, healthcare, staff

## Abstract

The novel corona virus disease COVID-19 was first diagnosed in humans in Wuhan, China in December 2019. Since then it had become a global pandemic. Such a pandemic leads to short- and long-term mental health burden for healthcare workers. Recent surveys suggest that rates of psychological stress, depression, anxiety, and insomnia and will be high for this group. Numerous organizations have since released guidance on how both healthcare workers and the general public can manage the mental health burden. However, these recommendations focus on specific healthcare workers (e.g., nurses or psychologists), are often not evidence-based, and typically do not situate guidance within a phased model that recognizes countries are at different stages of the COVID-19 pandemic. In this perspective paper we propose a phased model of mental health burden and responses. Building on work by the Intensive Care Society and the Royal College of Psychiatrists in the United Kingdom, we present a model that demonstrates how both staff and organizations might respond to the likely stressors that might occur at preparation-, pre-, initial and core-, and longer-term-phases of the pandemic. Staff within countries at different stages of the COVID-19 pandemic will be able to use this model. We suggest practical tips for both healthcare workers and organizations and embed this within up-to-date scientific literature. The phased model of mental health burden and responses can be a helpful guide for both staff and organizations operating at different stages of the pandemic.

## Introduction

This paper aims to collate some of the current guidance on maintaining mental health during the COVID-19 pandemic, with a particular focus on frontline healthcare workers. It situates these recommendations within a phased model of mental health burden and responses, which builds off the work by the Intensive Care Society (2020) and the Royal College of Psychiatrists (Williams et al., 2020). We suggest this model demonstrates how both staff and organizations might respond to the likely stressors that might occur at preparation-, pre-, initial and core-, and longer-term-phases of the pandemic. These recommendations are situated within relevant psychological literature, and derived from the clinical experiences of two of the authors, GL and BD-W. This text is primarily aimed at frontline staff and managers working in healthcare settings.

First, we describe sources of mental health burden for staff. Then we briefly highlight experiences of Chinese staff and interventions implemented there, before moving on to list a range of possible psychosocial interventions and underscore some key principles that can be derived from these. Finally, we present the phased model of mental health burden and responses.

## Background

The novel corona virus disease COVID-19 was first diagnosed in humans in Wuhan, China in December 2019 (World Health Organization [WHO], 2020a). The disease is found in individuals infected by the severe acute respiratory syndrome coronavirus 2 (SARS-CoV-2). The coronavirus can be transmitted between people via droplets, typically in coughs and sneezes. This can occur directly between people or indirectly by touching one’s mouth, nose or eyes. SARS-CoV-2 has spread rapidly across the globe and in March, 2020 the World Health organization (WHO) classified the outbreak a pandemic (World Health Organization [WHO], 2020b). As of July 29, 2020, there were 16,341,920 recorded cases and 650,805 fatalities globally (World Health Organization [WHO], 2020c).

Such a pandemic leads to short- and long-term mental health burden for healthcare workers. Emerging, current literature suggests that psychological distress is a very real outcome for staff providing health care amidst the COVID-19 pandemic. A study published March 23, 2020, surveyed 1257 healthcare workers in 34 hospitals in China (Lai et al., 2020). It found that rates of psychological stress were high: 50.4% had symptoms of depression, 44.6% for anxiety, 34% for insomnia, and 71.5% for general psychological distress^1^. Nurses, female staff, staff in Wuhan, and staff working directly with patients were more likely to have “severe” scores on these outcomes.

These findings are not unique to COVID-19. Studies into the SARS outbreak in 2003 reported psychological symptoms in 89% of workers in high-risk situations (Lai et al., 2020). This is all the more understandable given one in five SARS infection cases were healthcare workers (Chan-Yeung, 2004). Long-term psychological distress can result from the psychological stress experienced during such a pandemic (Lai et al., 2020). It is likely that the impact of stress associated with managing and providing care in uncertain and ever-changing circumstances may negatively impact on the immune system, weakening staff members’ ability to fight off the virus.

## Sources of Mental Health Burden for Staff

Currently the world is responding to an unprecedented pandemic and medical crisis that has not been seen for 100 years. Those working on the frontline are therefore exposed to a variety of sources of mental health burden which we outline below:

• Risk of contamination of the virus; compliance with biosecurity measures including constant vigilance, equipment use and isolation practices; tensions between patients and staff; and the stigmatization of healthcare workers coming into contact with patients with COVID-19 (International Federation of Red Cross and Red Crescent Societies, 2020).
• Abnormal mourning for the death of a loved one, home quarantine and social isolation, disruptions to work routines, sensitivity to and obsession with cleanliness and hygiene, the closure of public and private institutions, rumors about the disease, and the loss of social capital (Javadi et al., 2020).
• Uncertainty. This leads to stress and anxiety (Shanafelt et al., 2020). Stress is higher where staff have high work demands (heavy workload, time pressure, periods of intense concentration) but low work control (low levels of autonomy and decision-making input). Motivation and performance are lower when stressors are perceived as hindrances. Examples of hindrances include: unclear objectives, conflicting requests, red tape, organizational politics, and various other work-related hassles (Bolino, 2020).
• Weakened immune system due to high levels of stress (Segerstrom and Miller, 2004).
• Staff inquiries, physical exhaustion, sleep disruption, and fear and emotional disturbances (Li et al., 2020).
• Staff not knowing they can go home if they are ill or can work from home where appropriate (Beckman et al., 2020).
• Feeling vulnerable, loss of control, concerns about health of self and others, changes in working patterns/routine, feelings of personal danger, being isolated, lacking necessary supplies to conduct their work (Lai et al., 2020).
• Redeployment of the clinical workforce will be challenging. Clinicians are expected to work within unfamiliar territory, often with new teams/people, new processes, clinical procedures and equipment. Additionally clinicians are being released from their pre-registration studies early to contribute and work within frontline services (Royal College of Nursing, 2020).
• Implicit and explicit racism toward staff of Chinese origin (The Guardian, 2020).
• Abuse from detained patients including verbal insults intended to hurt staff members to “share the pain” of isolation from families (personal communication with an advanced nurse practitioner, United Kingdom).
• Pre-existing mental health vulnerability including previous trauma and mental ill health (Mental Health Foundation, 2020).

Having identified some of the sources of mental health burden in staff, this document describes principles that should underpin how hospitals and healthcare organizations can implement psychosocial interventions and organizational practices to mitigate these.

## Experiences From China

Some of the hospitals in China that were most affected by COVID-19 implemented a three-pronged approach to care for the mental health needs of staff:

1. Psychological intervention medical team to develop online courses to manage common psychological problems.
2. Psychological assistance hotline team to offer guidance and supervision to callers to help solve psychological problems.
3. Individual and group psychological interventions, including activities to release stress (Chen et al., 2020).

However, staff were hesitant to engage in these. Interviews with staff suggested that this reticence was due to a lack of immediate concern about being infected and feeling they did not need psychological support. They stated they needed more rest and personal protective supplies, and that they wanted mental health training or mental health staff to assist them when interacting with difficult or aggressive patients.

Revised interventions were implemented. Hospitals provided space for staff to rest and isolate themselves from families; staff were provided food and daily living supplies. New staff were trained in how to interact with difficult or aggressive patients; security teams were engaged if necessary. Detailed rules on appropriate use of personal protective supplies were written. Hospitals also established leisure activities; gave training to staff on how to relax properly; and counselors were embedded into the workplace to listen to staff and provide necessary help (Chen et al., 2020).

## How Can Staff and Organizations Respond to Different Phases of the COVID-19 Pandemic?

The Intensive Care Society (United Kingdom) offers several helpful ways of thinking about maintaining staff mental health before, during and after the COVID-19 pandemic (Intensive Care Society, 2020). Hospitals should think about where their organization is in relation to phases of the pandemic, be cognizant of the issues and impacts these will likely have for them and take note of the recommended approaches to these phases.

We have expanded on this guidance by incorporating mental health expertise to provide further context to their recommendations, practical tips for organizations and for individual staff. Subtle changes have been made to the phases outlined by the intensive care society as we have added in a preparation phase and combined the initial and core phases and the end and long-term phases acknowledging also that although these phases are linear, the overall process is cyclical and not rigid or fixed. We have added a preparation phase as different organizations and countries are encountering this pandemic at varying points; however, many international healthcare providers may well have passed this point now.

**FIGURE 1:**
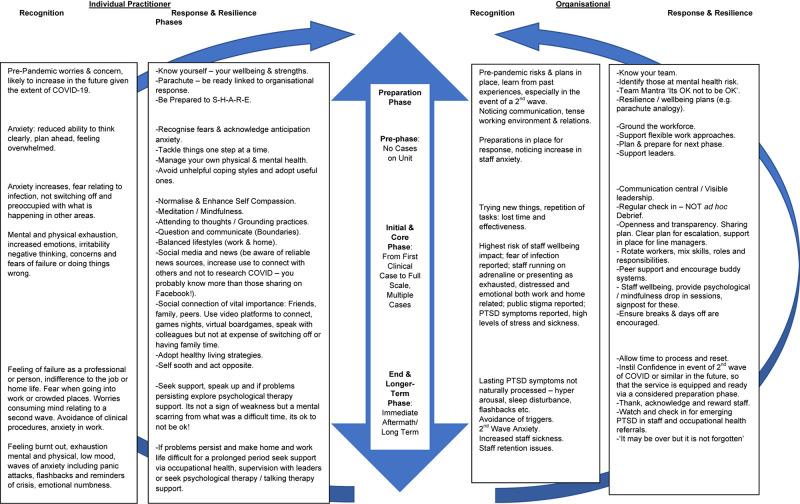
Phased model of advice for recognising and responding to mental health burdens during the COVID-19 pandemic.

Our aim is to offer guidance providing practical mental health support and advice to a range of frontline staff and organizations internationally who are working on the front line of this global pandemic. The guidance is written alert to the concern that services may face a 2nd wave or future pandemic. In Figure 1 we provide a flow chart that gives an overview of the phased advice and practical tips. More detail for each phase will be described within text.

### Preparation Phase

#### Individual Responses and Building Resilience

We have identified the need within the preparation phase for self-reflection, knowing your own needs and strengths and sharing them with someone you can trust to plan and prepare. There is a need to have a personal understanding of our triggers for stress as well as personal coping strategies for managing distress. As the team develops and membership evolves, time should be taken to discuss wellbeing and self-care routinely in the short-term and alongside supervision in the long-term. Be prepared to “Share”:

• **S**ee it?
• **H**ear it?
• **A**re you feeling it?
• **R**eport it and let someone know.
• **E**mbrace your needs and be a model for others to share.

#### Organizational Response

Organizationally, leaders are required to understand the needs of their workforce and establish if any members of the team may be more vulnerable than others to mental health difficulties including:

• Those with existing needs or current mental health difficulties.
• Those who have caring responsibilities in their home lives.
• Those who may have recently survived a stress or trauma experience.

Identification in the preparation phase will not identify everyone who might experience challenges to their mental wellbeing during the pandemic. However, it will enable teams to identify those most vulnerable so that plans can be put in place to support them. Buddy systems where peer support can be provided should be considered, as should a common compassionate mantra of “It’s ok not to be ok” due to the stigma often associated with mental health difficulties (Highfield et al., 2020; Stuart, 2016).

Resilience and wellbeing plans for staff should be encouraged. These should recognize the stressors that can present day to day in healthcare but particularly how this is magnified in such instances as this. These plans, written by team leaders, should describe triggers for stress, how one presently copes, early signs of distress (change to baseline), how they or team members can help. Wellbeing plans could include a parachute analogy, where leaders develop a plan like the weaving of a parachute that is there to soften and protect people in events of crisis such as this, instead of waiting until it is too late, therefore adopting a proactive rather than reactive approach. Support staff to make new mental health disclosures as the outbreak may bring these to the fore (Mental Health Foundation, 2020).

### Pre-phase

#### Individual Responses and Building Resilience

We advise that there is a need for people to recognize that fears and anxieties are justified and it is natural for these to be present in the face of threat as these fears enable us to identify risk and keep safe (World Health Organization [WHO], 2020b). Sometimes the anticipation of stressful events can be worse than when the actual event occurs. During the actual event we might neglect our emotional needs by focusing solely on our current tasks. During the pre-phase we have lots of time to think about what may occur, how it may feel, worst case scenarios, and what the job will be like in the initial and core phase.

Instead we suggest that whilst organizational preparations are made, individuals should tackle one task at a time, trying not to become preoccupied with future threats that cannot yet be addressed. The whole picture can be overwhelming (Williams et al., 2020). Instead we advise that your focus should be on making sure you are managing your own mental well-being. This is as important as your physical health is for tackling challenges that may present. A worldwide pandemic is an unprecedented scenario – identify and use strategies and positive coping techniques that you have used previously that have worked for you. However, avoid unhelpful coping such as smoking or drinking alcohol.

#### Organizational Response

At this pre-stage, team grounding is important. Grounding involves noting the emotional and cognitive information being shared in a group, acknowledging this and using it to structure an agenda for discussion. This is important because thoughts and emotions can become amplified within a group setting and fear and anxiety can migrate across team members (Smith and Mackie, 2015; Weisbuch and Ambady, 2008). Therefore, in the same way that we ask a client to ground themselves to the present when their distress exceeds their window of tolerance, the team leader may need to offer a greater sense of present moment awareness. A number of techniques are applicable with groups. For instance, ask the group to clap their hands at the same time or stamp their feet. Hold regular team meetings (making use of virtual tools where necessary) (International Federation of Red Cross and Red Crescent Societies, 2020).

One might also offer realistic reassurance – encourage team openness via adoption of the mantra “its ok not to be ok.” Consider what communication will look like for each team whilst remaining aware of the team’s current needs. Ensure you think about protected characteristics of staff i.e., do measures affect all staff equally? (Mental Health Foundation, 2020). Offer flexible working routines for staff personally affected by the virus e.g., illness or death in the family, childcare duties (World Health Organization [WHO], 2020b). Ensure that managers are also considerate of their own individual needs and they are themselves not immune to the mental wellbeing impact such events have due to the high levels of stress they will also be enduring. Part of this is sharing stories with other managers and team leaders (Mental Health Foundation, 2020). Unlike the individual response, the organizational response will require longer term planning in order to respond effectively to worst case scenarios, i.e., access to beds, equipment including PPE, and resources redistribution.

### Initial and Core Phases

#### Individual Responses and Building Resilience

This phase has been identified as the highest psychological risk phase (Highfield et al., 2020). In Figure 1 we provide some practical ideas which in this phase are going to be of paramount importance.

• *Enhance Self Compassion* – We can in times of high stress and emotional extremes often become critical of ourselves or our performance. Be compassionate. How would you speak to your friend if they were feeling this way? What advice would you give? How would you hold yourself or hold them? Now speak to yourself in the same way … say the same things. Use a mantra: “it is fine to feel like this” (Irons and Beaumont, 2017). Identify activities that help you self-sooth that you can still engage within the comfort of your own home. The tasks you never got around to completing, the film you’ve been wanting to watch, etc.
• *Mindfulness* – Mindfulness is the practice of being in the present moment, on purpose, taking a non-judgmental stance and is underpinned by mediation practices (Kabat-Zinn, 2013). Mindfulness practices to manage our stress and emotion are becoming increasingly popular practice. A variety of Apps including Headspace can be purchased for mobile devices, and providers such as YouTube include narrative examples of mindfulness.
• *Grounding –* It is important to take stock of what is going on around you and ground back to what is happening in that moment. Grounding techniques can be used to help people stabilize in the face of trauma, stress, and dissociation (Foureur et al., 2013). Some useful techniques include placing both feet into the ground and stomping, clapping hands, or looking around the environment to name and describe three objects you can see or three sounds you can hear, hence using the senses to assist in grounding.
• STOP, GROUND, BREATH is another strategy in which we encourage you to use your breath to as a grounding technique such as, breath in through the nose and out of the mouth … breath in (2 s), hold two and breath out completely (take three breaths).
• *Balance home and work* – Try to distinguish the two by reducing time spent watching the news, focusing on things away from COVID-19. Taking a break at home is important as work will be dominated by the pandemic.
• *Social Media* – Use credible sources, keep in touch with friends and family but choose what to read and engage in. “Sandra” on Facebook probably knows much less than you, so do not let her posts further impact on your emotions.
• *Social Connection* – Connect with friends, family, peers. Recent surveys of the United Kingdom general public found this to be one of the most helpful coping mechanisms (Holmes et al., 2020). Use video to see faces. Engage in virtual games nights and board games. Social connectedness with people experiencing the same difficulties is important. Use buddy systems, check in on each other but balance this with family and no-work downtime (Williams et al., 2020).
• *Adopt healthy living strategies* – These will reduce your emotional vulnerability and make you more able to manage you own stress and emotions (1) Take care of physical health and treat physical illness, (2) balance eating; low mood often results in reduced appetite or comfort eating which in the short term might feel helpful but longer term make you feel worse, (3) avoid mood altering drugs (including alcohol), (4) sleep well; we all require rest especially in times of stress and high anxiety which alone can be exhausting, (5) engage in exercise; physical fitness and a release of pressures are essential, and (6) build mastery by finding activities that provide you with a sense of accomplishment (Linehan, 2014).
• *Routine* – Maintain a routine as much as possible. Write a list of the things you would like to do around the house that can now be achieved in your out of work time but balance this with relaxation time.
• *Act Opposite* – Don’t watch too much news, programs, and films related to the current challenges or sad themes; act opposite and watch comedic, upbeat or enlightening programs and films. Don’t listen to music that makes you sad or upset; listen to upbeat songs. Don’t withdraw and isolate from those you love; use this as a chance to reconnect and learn new things about people (Linehan, 2014).

#### Organizational Response

During this stage, communication is going to be essential. Provide timely, accurate and evidence-based information on the virus and the hospital’s response, including worse case scenarios (International Federation of Red Cross and Red Crescent Societies, 2020; Mental Health Foundation, 2020; World Health Organization [WHO], 2020b). Ensure present, visible and easily recognized leadership is present. Be a role model for how you would expect staff to behave (personal health and wellbeing, appropriate use of personal protective equipment) (World Health Organization [WHO], 2020b). Ensure regular communications are provided, with the opportunity for regular check in and discussions. Frame/describe the hospital’s response to COVID-19 as a challenge from which staff can all grow and develop; do not describe it as a hindrance (Bolino, 2020). Give staff autonomy and input into decision-making where possible (Bolino, 2020). Remove bureaucratic hindrances to flexible working, such as blocks on virtual meetings or remote working (Bolino, 2020).

Do not perform psychological debrief as this is not advised during traumatic events and can make things worse (National Institute for Health and Care Excellence, 2018). Engage the workforce in peer support and buddying practices and within this consider partnering experienced people up with those who may be less experienced or new (International Federation of Red Cross and Red Crescent Societies, 2020; World Health Organization [WHO], 2020b). Adopt a mantra and compassionate response to staff in that “its ok not to be ok” and allow for opportunity for people to discuss their own needs, concerns and feelings. Signpost to psychological first aiders and drop-in sessions for staff support, you might even assign a single member of staff as the representative on this (International Federation of Red Cross and Red Crescent Societies, 2020; Mental Health Foundation, 2020). Ensure, positively monitor, and encourage work breaks (World Health Organization [WHO], 2020b). Mindfulness practices within the workplace can also prove beneficial, with programs of mindfulness-based stress reduction provided to support health care professionals, producing positive results (Irving et al., 2009).

### End and Longer-Term Phase

#### Individual Response and Building Resilience

Once the COVID-19 pandemic has passed things are unlikely to return to normal. You will be likely reflecting on what has occurred and your responses to this. Make sure you stay connected with colleagues and that you share your experiences. Feeling distressed after your experience is normal and understandable. This is all the more likely if you have been moved into a new role or redeployed into a new working environment where routines, rules and colleagues are unfamiliar. The Adaptive Information Processing model (AIP) proposes that new information taken into the brain through our senses is assimilated into existing memory networks. This allows us to make sense of this information when we recall this information in the future. It is important to give yourself time to process experiences into your existing cognitive structures (memory networks).

The latest guidance for the assessment and treatment of trauma proposes “watchful waiting” rather than psychological debriefing (National Institute for Health and Care Excellence, 2018). This is because many individuals exposed to trauma do not develop post-traumatic stress disorder (PTSD). Most people recover from the early experience of traumatic stress symptoms without formal intervention (Grey, 2009). However, a minority can develop symptoms and it important to recognize symptoms. The Diagnostic and Statistical Manual of Mental Disorders version 5 (DSM-V) refers to pre-, peri-, and post-factors that influence the risk of PTSD (including prior trauma, prior health needs, inappropriate coping strategies, and negative appraisal), and is a good source to consult (American Psychiatric Association, 2013). As such you should continue to use the strategies you have found work for you. Observe and notice changes in sleep, feeling unreal or feeling disconnected, re-experiencing things that have happened. Be aware if there are things you are avoiding in case they trigger negative emotions. Report any of these as you may need further support to your supervisors or supportive friends and family.

#### Organizational Response

Allow and expect that time is afforded for all in the team to process their experiences and reset. The crisis might be over in terms of immediate threat but the after-effects psychologically on the workforce may not instill confidence of readiness and lessons learnt if fears of a second wave are prevalent but ensure in the background the organization is ready for such an occurrence. Look to thank, acknowledge, and reward the workforce. Reflect on the lessons learnt using a known model of reflection, such as Description, Feelings, Evaluation, Analysis, Conclusion, Action plan (Gibbs, 1988). Take a watchful waiting approach and check in for any emerging symptoms of PTSD in staff, making sure appropriate referrals are made. Adopt a stance of “It may be over but it is not forgotten.” Continue regular communications with staff following shifts to check in and see if anyone requires further support. The UK National Institute for Health and Care Excellence (2018) guidelines on the treatment and management of PTSD suggest looking out for the following signs:

• Hyper arousal;
• Sleep disturbance;
• Flashbacks or re-experiencing;
• Avoidance of triggers.

If staff present with any of these offer them direction to support services or simply propose a talk in protected time.

## Conclusion

There is plenty that hospitals and healthcare providers can do to help healthcare staff manage mental health burden. Early experiences from China and more recently in Europe suggest that healthcare staff will likely experience negative mental health outcomes due to the pandemic and their employment. This paper is a guide to managing the mental health burden of the clinical workforce in an attempt to support their mental wellbeing and organizational responses. The phased model of mental health burden and responses can be a helpful guide for both staff and organizations operating at different stages of the COVID-19 pandemic. Organizations and individuals implementing this model in whole or in part should also consider undertaking a suitably powered evaluation of both staff and organizational outcomes. This would help to develop a body of evidence that supports embedding the model in routine practice or making signposting alterations.

## Data Availability Statement

The original contributions presented in the study are included in the article/supplementary material, further inquiries can be directed to the corresponding author.

## Author Contributions

All authors listed have made a substantial, direct and intellectual contribution to the work, and approved it for publication.

## Conflict of Interest

The authors declare that the research was conducted in the absence of any commercial or financial relationships that could be construed as a potential conflict of interest.

## Footnotes

1 Depression (defined as a total score of ≥5 in the Patient Health Questionnaire-9); anxiety (defined as a total score of ≥5 in the Generalized Anxiety Disorder-7); insomnia (defined as a total score of ≥8 in the Insomnia Severity Index); stress related symptoms (defined as a total score of ≥9 in the Impact of Events Scale-Revised).
